# Partial (incomplete) removal of granulation tissue using modified minimally invasive surgical technique in treatment of infrabony defects (randomized control clinical trial)

**DOI:** 10.1186/s12893-024-02509-w

**Published:** 2024-08-12

**Authors:** Ahmed Adel Ibrahim, Omnia Khaled Tawfik, Hani ElNahass

**Affiliations:** 1 Faculty of Dentistry, Cairo University, 5 Mostasmr Al sagheer st, Sheikh Zayed, Giza, Egypt; 2 Periodontology department, Cairo University, Giza, Egypt

**Keywords:** Granulation tissue, Infrabony, Osseous defect, Periodontitis, Regeneration

## Abstract

**Aim:**

This study aims to compare the clinical and radiographic outcomes after complete versus incomplete removal of granulation tissue (GT) during modified minimally invasive surgical technique (M-MIST) for management of periodontitis patients with deep pockets associated with infra-bony defects.

**Methodology:**

Ten patients with a total of 14 deep non-resolving pockets (≥ 5 mm) associated with a vertical infra-bony defect were recruited for this study. They were randomized into 2 groups; a test group with incomplete removal of GT and a control group with complete removal of GT. Clinical parameters of clinical attachment level (CAL), residual probing depth (rPD) and buccal recession (Rec.) were recorded every 3 months. Radiographic periapicals were taken at baseline, 6 and 9 months. The significance level was set to 0.05.

**Results:**

None of the results showed statistical significance between the 2 groups (*p* > 0.05). The test group showed less CAL gain (2 ± 0.87 mm, *p* = 0.062), more reduction in rPD (3.1 ± 0.96 mm, *p* = 0.017) and more recession (0.857 ± 0.26 mm, *p* = 0.017) than control group CAL gain (2.4 ± 0.58 mm, *p* = 0.009), rPD reduction (2.9 ± 0.3 mm, *p* = 0.001) and recession (0.5 ± 0.34 mm, *p* = 0.203) respectively. Control group had linear reduction in depth defect (DD) (0.68 ± 0.287, *p* = 0.064) compared to an increase in DD in test group (-0.59 ± 0.5, *p* = 0.914).

**Conclusions:**

No statistical significance were observed in healing parameters between complete removal of GT in M-MIST and incomplete (partial) removal of GT of deep pockets with infra-bony defects both clinically and radiographically. Further studies with larger samples are needed to confirm the results.

## Introduction

To date, periodontitis remains one of the most common diseases affecting dentition [1–3] It is caused by both microbial-associated and host mediated inflammatory reactions resulting in damage to the supporting structures [3]. It may present clinically in many forms among them are deep pockets and infra-bony defects. These common sequalae are challenging to treat and make simple non-surgical treatment insufficient [4] Surgical treatment modalities are better suited to treat deep pockets with infrabony component where the treatment aims to reconstruct the attachment apparatus while preserving of the pre-surgical soft tissue contour and height [5] This is especially true with minimally invasive surgical techniques and their modifications (M-MIST), where an improvement in healing and preservation of periodontal tissues was achieved simultaneously minimizing the surgical trauma [6, 7].

Access flaps, mechanical debridement of root surface and removal of the granulation Tissue (GT) residing in the bony defects has been considered to be conventional steps in every periodontal surgery. The time consuming process of complete removal GT removal, is considered an integral part of the healing process as it represents the chronic constituent of the periodontal defect [8, 9]. Its thorough and complete removal is routinely performed in all periodontal surgeries to ensure optimum healing [7, 10] although Lindhe & Nyman, ( [10]) reported that the complete removal was not considered necessary for healing. Nonetheless, GT has continued to routinely be eliminated from all periodontal defects [7, 10].

In a study by Ronay [11], the infected GT extracted surgically from infra-bony defects was analyzed and shown to contain stem cells with embryonic markers. Similarly, other studies showed that granulation tissue contained multi-potent stem cells with osteogenic potential that could be used for auto-transplantation in other defective sites with regenerative potentials similar to cells obtained from healthy GT [12, 13]. Stem cells isolated from infected GT also revealed higher migratory properties than cells obtained from healthy GT [14] This inflamed GT is simpler and easier to obtain and can potentially be a source for these progenitor cells. Recently, a systematic review and meta-analysis concluded that periodontal GT acts as a source of putative MSCs (mesenchymal stem cells). When considering that inflamed GT is plenty, it can be a viable source for progenitor cells as it is simpler and easier to obtain [15]. These cells have a special therapeutic potential in regenerative therapy where true regeneration is considered incredibly complex [8].

An array of factors guide the wound healing process: According to Melcher, the nature of the attachment established between the tooth and the periodontal tissue depends on the type of cells that repopulate the wound adjacent to the root surface [16]. Furthermore, the shape and size of the defect and the stability of the blood clot within the space also determine the degree of regeneration that can be achieved [9, 17]. Incomplete removal of GT provides less wound space, less defect size and more blood clot stabilization. With GT acting as a source of progenitor osteogenic cells when combined with minimally invasive techniques, it may improve hypothetically clinical outcomes and provide the required tenant of regeneration, a source of stem cells, a suitable scaffold markers [8].

The aim of the current study to test and compare the clinical and radiographic outcomes of complete versus incomplete (partial) removal of GT using M-MIST in management of deep pockets associated with infra-bony defects to assess their regenerative potential in terms of clinical attachment loss, probing depth recession, and radiographic bone fill in addition to patient reported outcomes.

## Subjects and methods

### Study participants

The study population for the current study were recruited from the postgraduate clinics of the department of Periodontology and Oral Medicine, Faculty of Dentistry – Cairo University. Recruitment started from December 2018 and to April 2021 until the target sample was reached. Healthy study subjects diagnosed with periodontitis with an age range of 25–55 years were enrolled in the study [18].

### Preoperative preparation

Non-surgical periodontal therapy was completed for all subjects, as an initial step. Supra and subgingival debridement was done using a combination of ultrasonic and hand instruments (Gracey curettes. Hu-friedy, USA). All study subjects were put on a strict oral hygiene program; brushing regularly in addition to chlorhexidine mouthwash (0.12%, Hexitol, Adco, Egypt) twice daily for 2 weeks with the use of interdental cleaning aids (flossing or interdental brush). After 6 weeks, patients were re-evaluated to check for non-resolving deep pockets using a 15 mm periodontal probe (UNC, Medsey, Italy).

Patients included in this study:

- were diagnosed with periodontitis (stage III or IV) grade B [19],
- having non-resolving residual pockets (≥ 5 mm) associated with vertical bone defect (≥ 2 mm), 6 weeks following non-surgical periodontal therapy [19].

Patients were excluded from the study if they have certain conditions:

- uncontrolled medical conditions [20],
- self-reported pregnancy or lactation [20],
- current smokers [21],
- grade II tooth mobility or higher [19].

These non-resolving residual pockets were randomized into 2 parallel groups using computer generated program. Allocation concealment was done using opaque concealed envelopes.

### Sample size calculation

The study sample size was calculated based on a study by (Mishra et al., 2013). Using a power of 90% and 5% significance level, 6 sites in each group were found to be sufficient to detect the difference between the 2 groups in CAL gain. This number was increased to 7 in each group to account for losses during follow up (20% more than the calculated). The sample size was calculated based on data from a study on the effect of M-MIST alone on the CAL assuming a difference of 1.5 mm between the means and a standard deviation of the difference in means of 0.7 mm. Sample size calculation was done using PS: Power and Sample Size Calculation software Version 3.1.2 (Vanderbilt University, Nashville, Tennessee, USA [18].

### Surgical method

Profound anesthesia was achieved using 4% Articaine hydrochloride/adrenaline anesthesia (Septanest, France) by infiltration labially or buccally at the defect site. Bone sounding was performed using a 15 mm UNC probe to determine the anatomy of the bony defect. A modified or simplified papilla preservation incision was performed according to the width of the papilla interdentally [19], If the width of the interdental space was ≤ 2 mm [22] or the modified papilla preservation technique at interdental sites ≥ 2 mm [23].After incising interdental papilla using 15c blades, a small labial or buccal sulcular incisions in the adjacent teeth were done for better accessibility. All loose interdental tissues underlying papilla at the crest of the alveolar bone was cut using micro-scissors. After that, muco-periosteal elevator was used to minimally elevate and reflect a full thickness flap buccally.

**Fig. 1:**
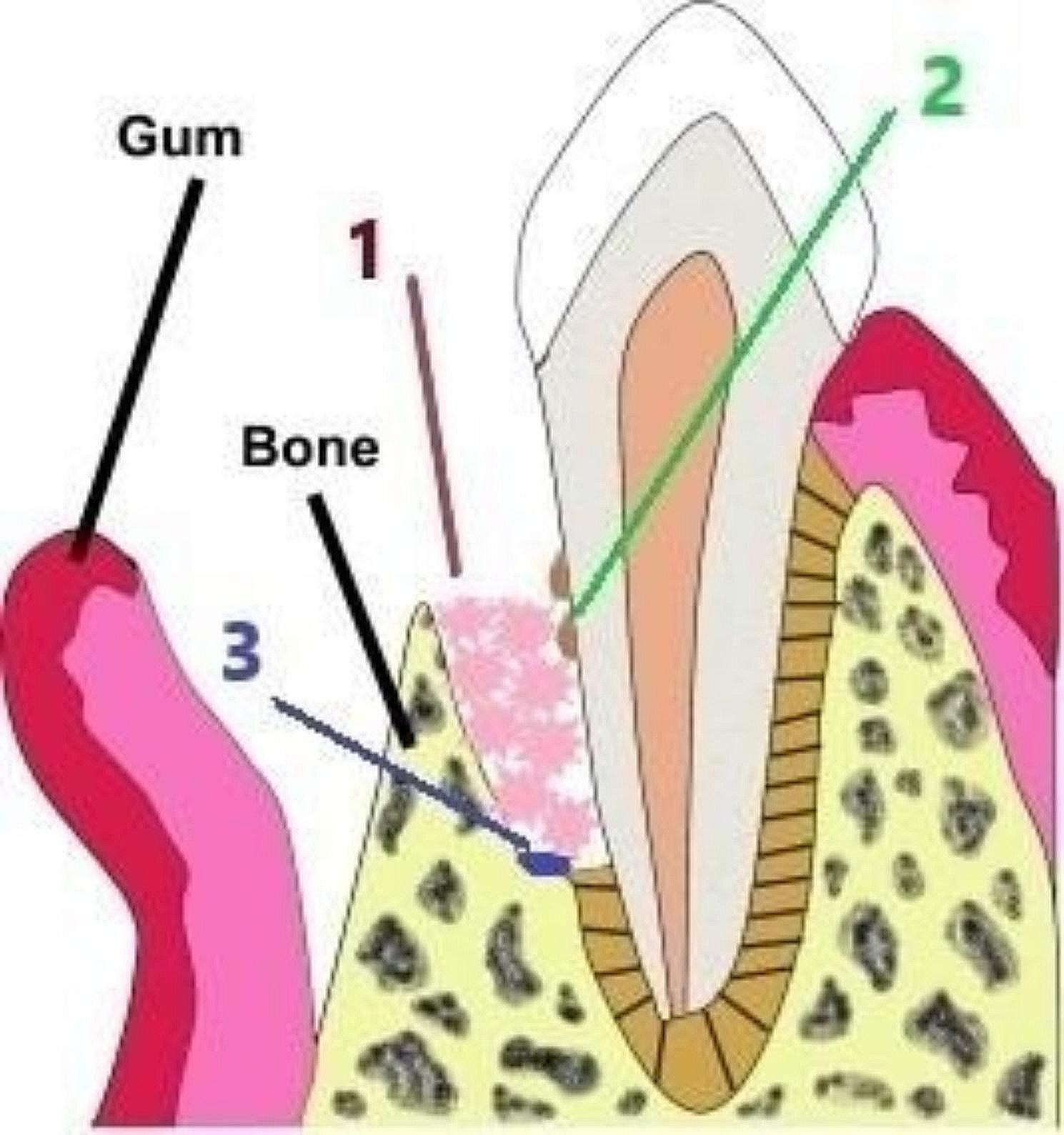
A diagram showing an infrabony defect components of (1) GT, (2) remaining deposits on root surface and (3) supra-crestal fibers at the base of the defect

**Fig. 2:**
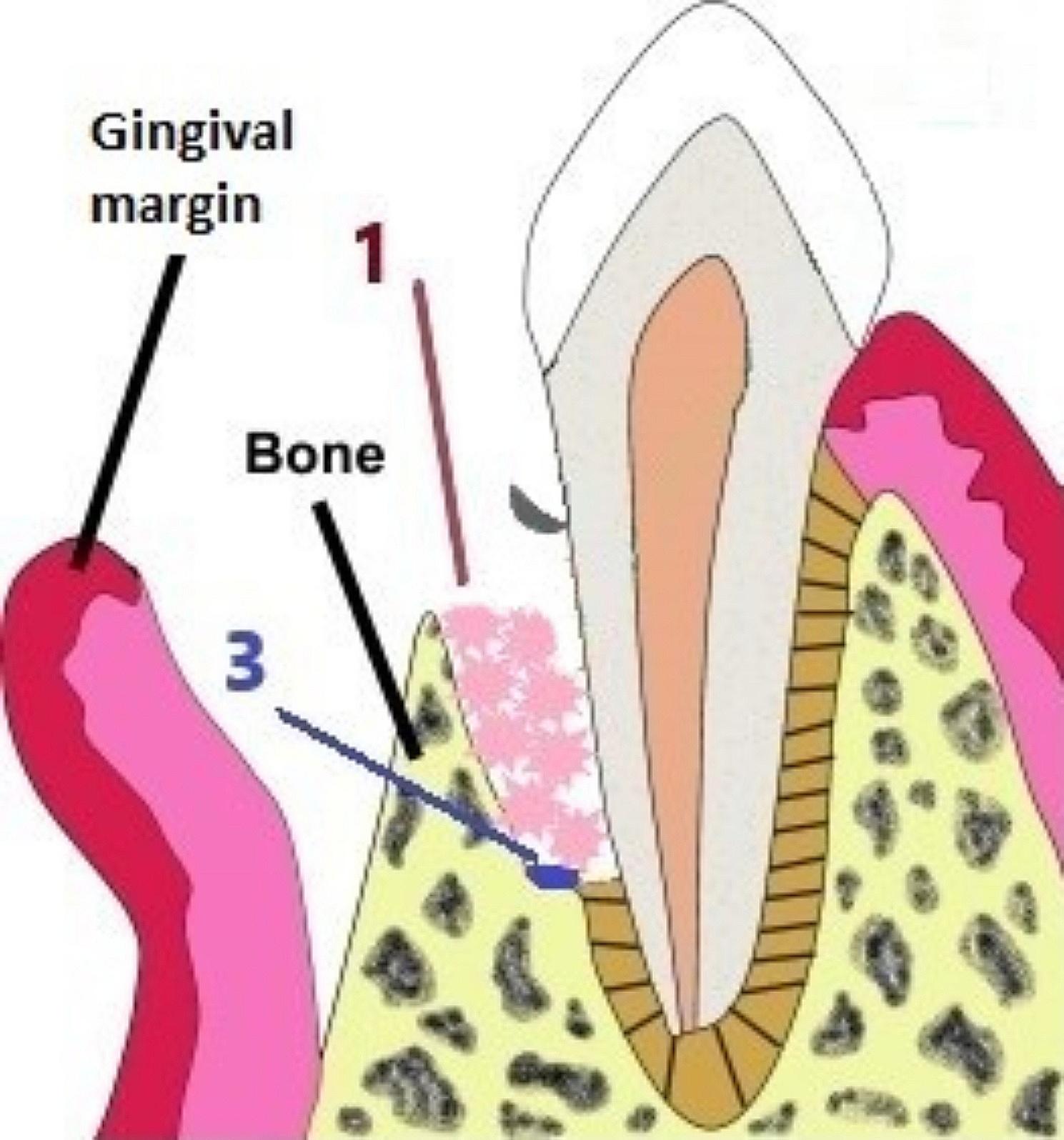
shows a diagram of an infrabony defect after root debridement with incomplete removal of GT

The root surface was debrided thoroughly by mini curettes (mini-five, Hu-friedy, USA). In the control group, the infrabony defect was cleared from all of the GT leaving behind a raw bleeding bone surface. In the test group, only unattached GT in the flap and beneath the interdental papilla was removed, leaving well formed (globular attached) GT in the defect site intact (Figs. 1 and 2). Finally, the surgical site was irrigated using sterile saline for both groups before suturing.

The wound was sutured by using a single modified internal vicryl suture (Laurell loop) using 6 − 0 or 7 − 0 suture material (Assut, Switzerland).

All surgical procedures were performed by the principal investigator (A.A.I) using loupes (Eighteeth 3.5x, China) for magnification, better vision and accessibility.

### Postoperative care

Ibuprofen 400 mg (Abbott, Egypt) was prescribed for pain control on a need basis for the first week postsurgically. Chlorhexidine mouthwash 0.12% (Hexitol, Adco, Egypt) was prescribed twice daily as a plaque control measure instead of tooth brushing in the surgical site for the first 4 weeks. After that, tooth brushing was resumed using a soft toothbrush in the surgical site.

**Fig. 3:**
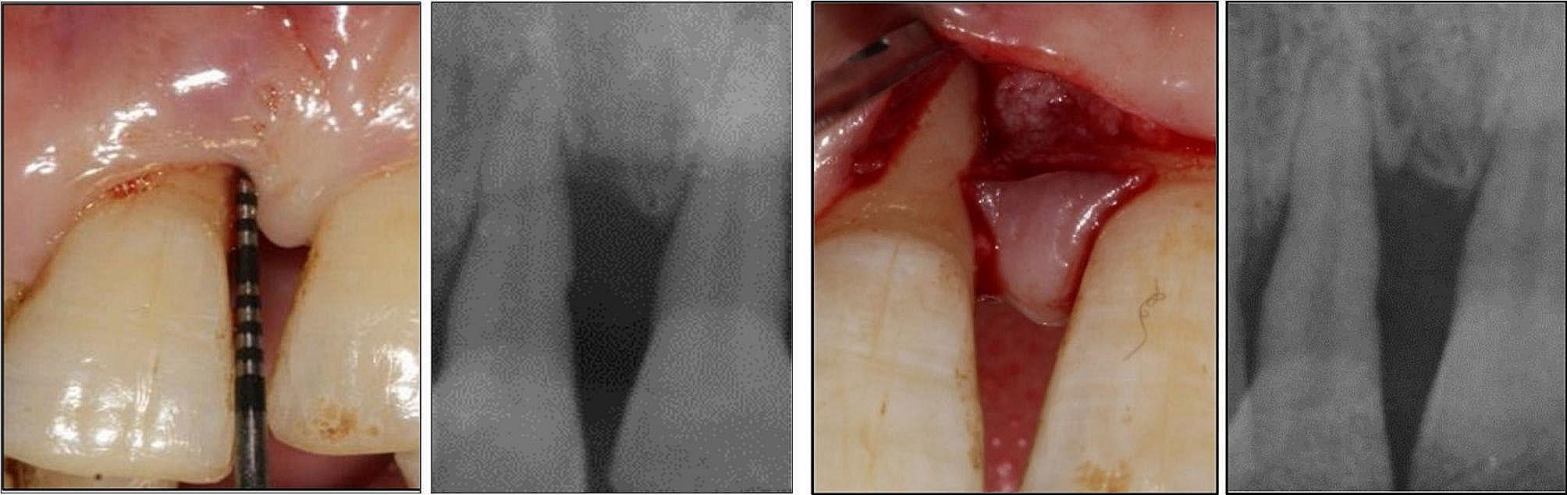
shows infrabony defect related to mesial surface of upper right central incisor (11) with rPD (8 mm) and recession ( 3 mm), radiographic images of the infrabony defect at surgical time and after 9 months follow up (control group)

**Fig. 4:**
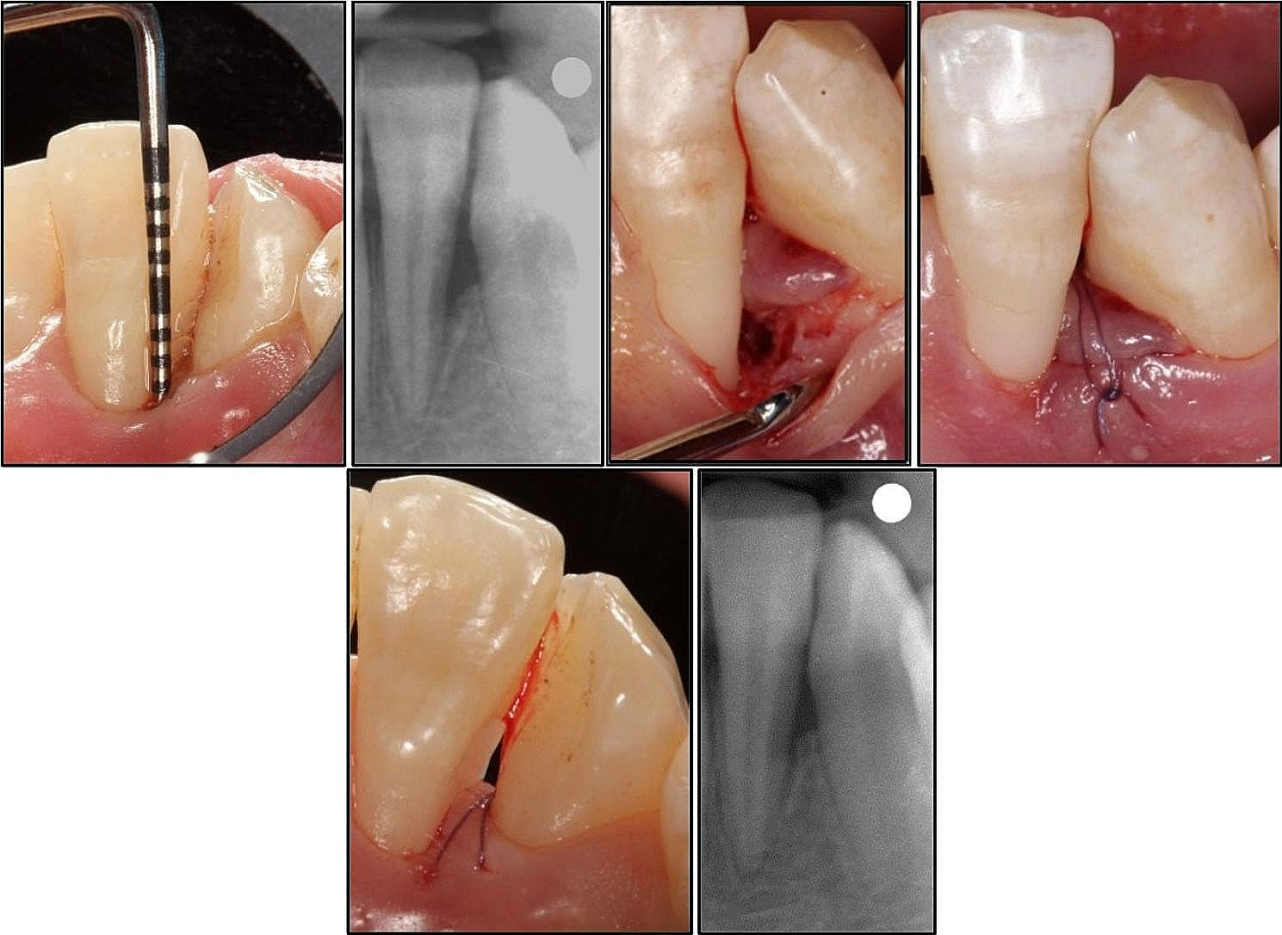
shows infrabony defect related to distal surface of lower left lateral incisor (32) with rPD (6 mm) and recession ( 2 mm), clinically at baseline, during surgical time and after suturing. Radiographs at baseline and in 9 months follow up (test group)

Sutures were removed 10 days post-surgically. Then, each patient was given oral hygiene instructions and recalled at 1, 2, 4 weeks then at 3, 6, 9 months intervals. Adverse events, if arose, were recorded at every visit. Gentle scaling with no subgingival instrumentation was performed during the last 3 visits Figs. 3 and 4.

### Clinical parameters

The primary outcome for the current study was the CAL (calculated from the sum of rPD and Rec. values). Secondary outcomes included: rPD, Rec., full-mouth plaque score (FMPS) and full-mouth bleeding score (FMBS). Clinical outcomes were recorded at baseline, 3, 6 and 9 months after surgery for each site by a blinded calibrated examiner (H.E.N.). All the measurements were taken by using a 15 mm periodontal probe (UNC, Medsey, Italy). All measurements were recorded to the nearest millimeter at the deepest location of the selected interproximal site.

### Radiographic parameters

Radiographic parameters were recorded at 0, 6 and 9 months: angle of the defect and defect depth (DD). Radiographic parameters were recorded using a size 2 Digora imaging plate in a film holder (Rinn XCP) for obtaining standardized digital paralleling periapical technique. All teeth were radiographed using “Mainray x-ray machine” (SOREDEX, Nahkelantie-Tuusula, Finland) with exposure parameters 70 kvp, 7 mA and exposure time of 0.08 s. After exposure, the DIGORA Optime laser scanner was used for scanning of the imaging plate and obtaining the digital image to be evaluated using DIGORA^®^ for Windows 2.7 (U.S.A).

From the scanned image a number of measurements were obtained. The defect angle was recorded by measuring the angle between the root surface and the surface of the bony defect as outlined by [24] Defect depth was measured linearly as the distance between the most apical part of the defect and one passing across the most crestal part of the alveolar bone [25]. The distance from the CEJ to the most crestal part of the alveolar bone was also monitored throughout the trial to measure crestal bone level stability.

All clinical and radiographic outcomes were reported by a calibrated assessor (Omnia) thus avoiding detection bias [26].

Postoperative pain were recorded using VAS scale. It was recorded by the patient 1 week postsurgical on the VAS scale and by reporting on the number of analgesic pills consumed during that period.

### Statistical analysis

Statistical analysis was performed using SPSS (Statistical package for the social sciences) version 20, IBM corp., U.S.A. Data were represented as mean and standard deviation. The Kolmogrov-Smirnov test was used to examine the normality of data distribution. Repeated measures analysis of variance (ANOVA) test was used to compare variables within each of the studied groups. If ANOVA were significant pairwise comparisons using Bonferroni correction was used to determine the source of variation. Independent sample student t-test was used to compare variables between the two studied groups. All tests results were considered statistically significant if the P- value was less than 0.05.

## Results

### **Study population** (base line characteristics)

**Fig. 5:**
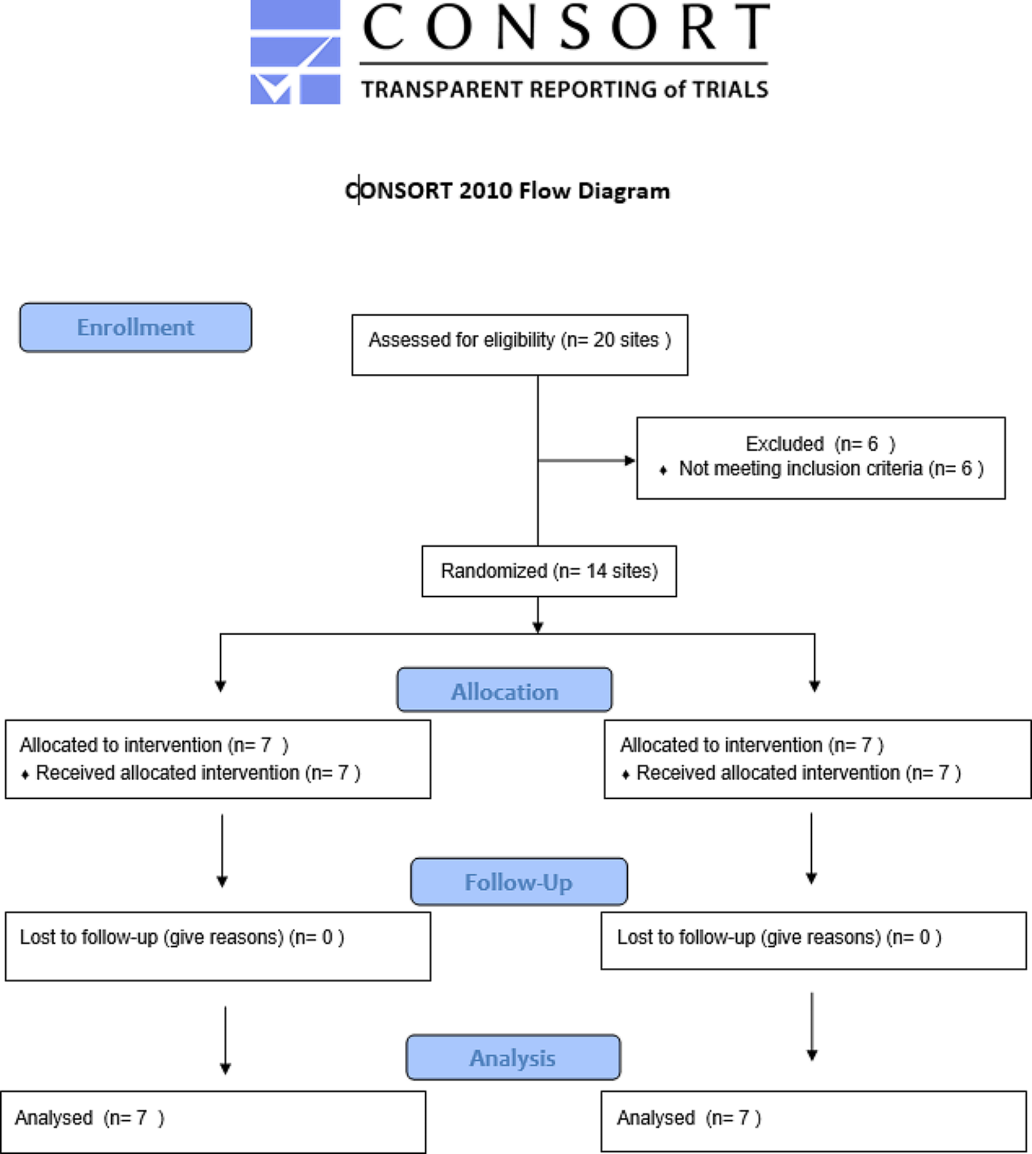
shows consort flow diagram of the current study

10 systemically healthy adult subjects with 14 eligible periodontal sites, 7 in each group, were included in the present study (Fig. 5). The study population consisted of 6 females and 4 males, with mean age at baseline of 38 years ± 4.19 in the control group and 37 years ± 3.95 in the test group (Table 1) both groups.

**Table 1:** Baseline characteristics: all data are expressed as mean and standard deviation

Demographic data	Test group (*n* = 7)	Control group (*n* = 7)	*P* value
Age	37 years ± 3.95	38 years ± 4.19	0.596
Gender (F/M)	(4/3)	(3/4)	
FMPS (%)	13.21 ± 1.77	12.63 ± 2.19	0.85
FMBS (%)	14.52 ± 0.54	13.84 ± 1.22	0.71
CAL (mm)	8 ± 2.08	7.08 ± 1.02	0.059
rRPD (mm)	7 ± 2.2	6.25 ± 0.76	0.241
REC. (mm)	1.28 ± 1.38	0.833 ± 1.3	0.809
BOP	0.571 ± 0.202	0.5 ± 0.342	0.844
**Defect characteristics**			
Angle of the defect (degree)	47.9 ± 19.67	46.3 ± 14.18	0.217
Defect depth (mm)	2.767 ± 2.3	2.22 ± 1.17	0.291
Distance from CEJ to BD	6.66 ± 1.68	6.52 ± 2.1	0.456
No. of remaining walls (n)	3 (2), combined (5)	3 (3), combined (4)	

Test group (incomplete removal of GT), Control group (complete removal of GT), FMPS (full-mouth plaque score), FMBS (full-mouth bleeding score), CAL, rPD, REC, BOP (bleeding on probing), CEJ (cemento enamel junction), BD (base of the defect), number of remaining walls were determined clinically

### 1-CAL

At baseline, there was less CAL in the control group (7.08 ± 1.02 mm) than in test group (8 ± 2.08 mm) however this difference was not statistically significant.

**Fig. 6:**
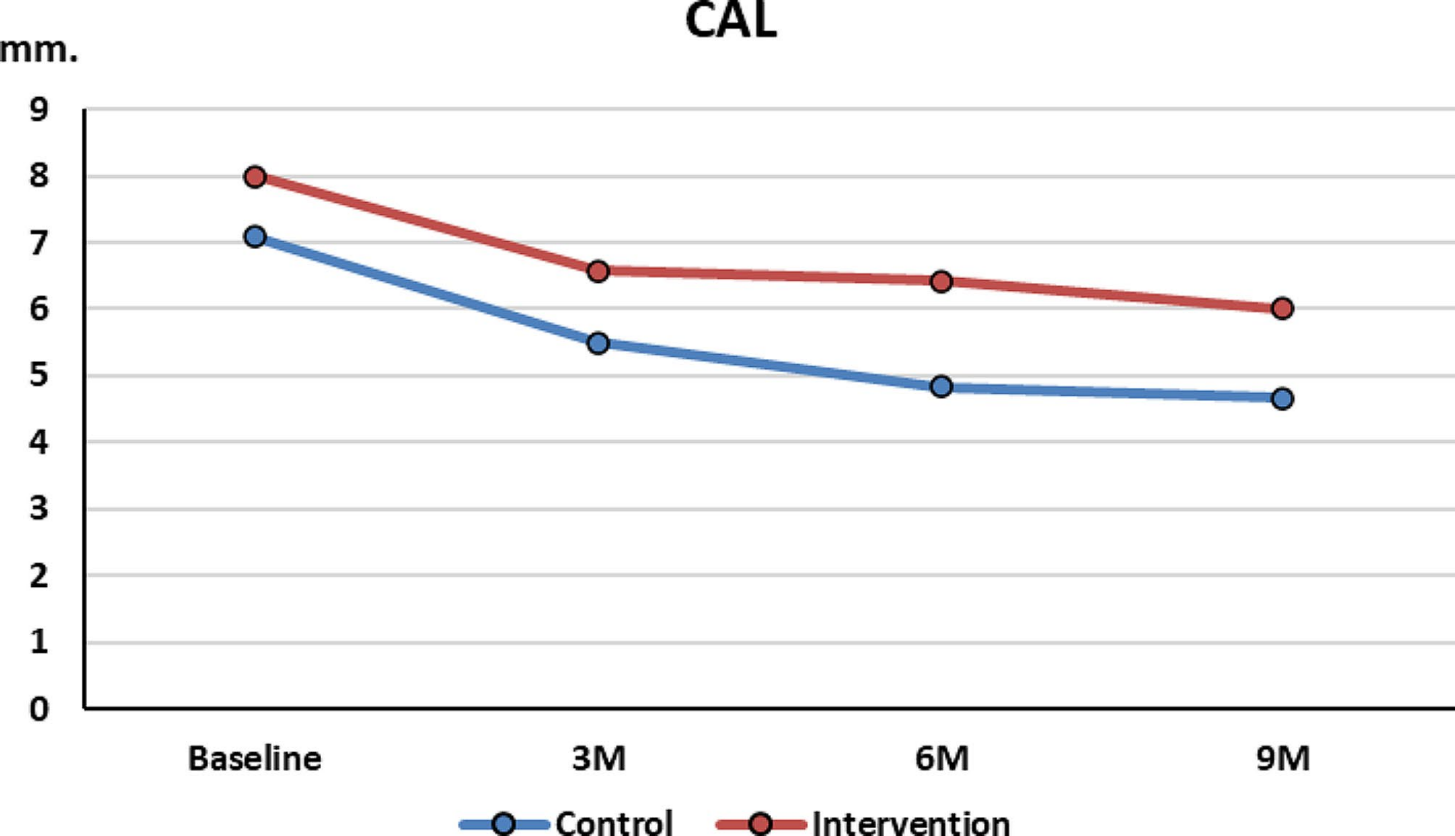
CAL change over time between the 2 groups

During follow up, the CAL gain at 3, 6 and 9 months in the control group was slightly higher than the test, however these differences were not statistically significant ( Fig. 6).

### 2-rPD measurement

At baseline, rPD of control group (6.25 ± 0.76 mm) was less than test group (7 ± 2.2 mm) however this difference was not significant. At 3 and 6 months, rPD reduction was noted in both groups with a slightly higher improvement in the control group. These changes were not statistically significant.

**Fig. 7:**
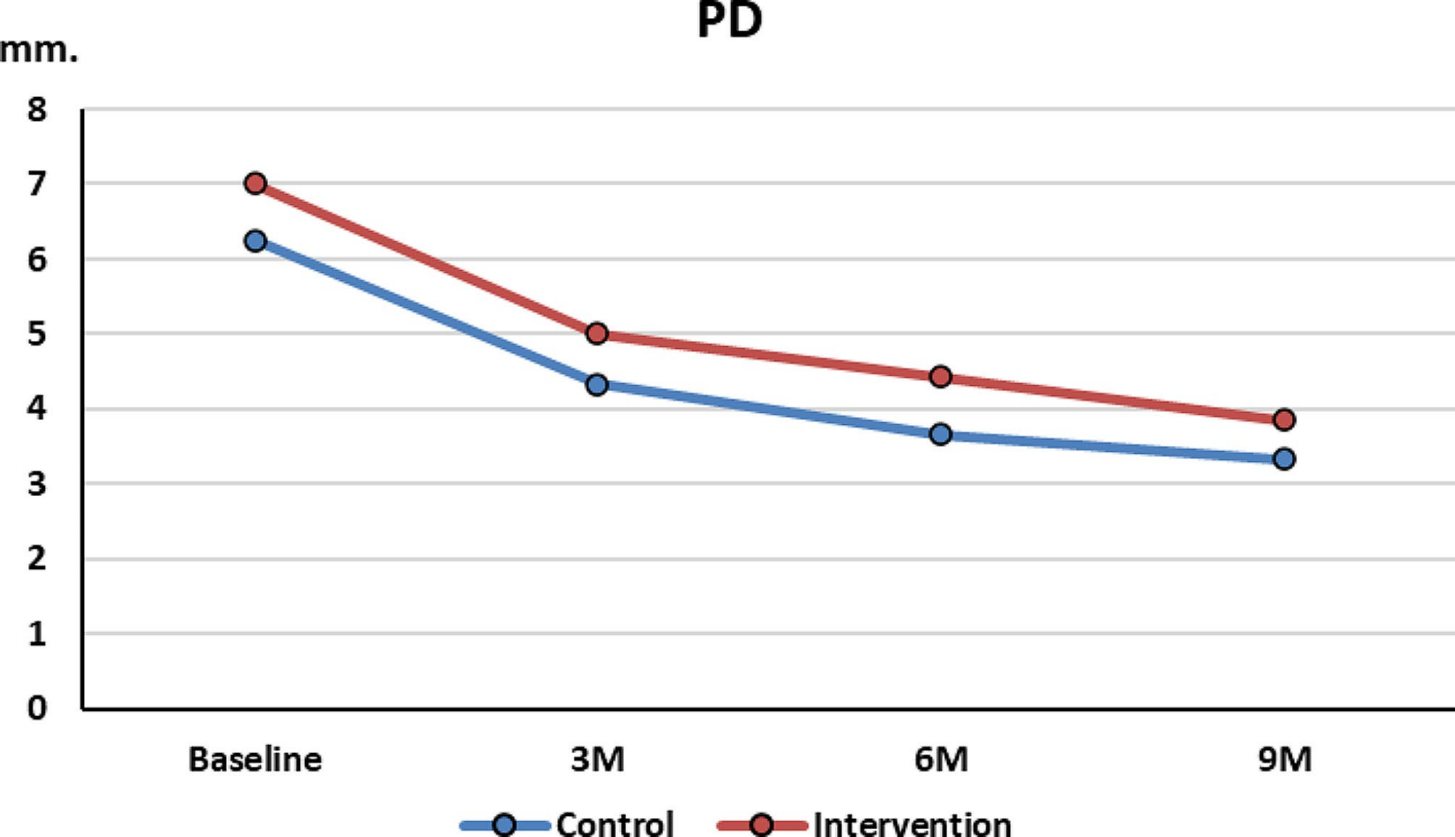
rPD reduction overtime between the 2 groups

At 9 months, comparable reduction in both control (2.9 ± 0.3 mm, *p* = 0.001) and test group (3.1 ± 0.96 mm, *p* = 0.017) (Fig. 7).

### 3- Rec. Measurement

**Fig. 8:**
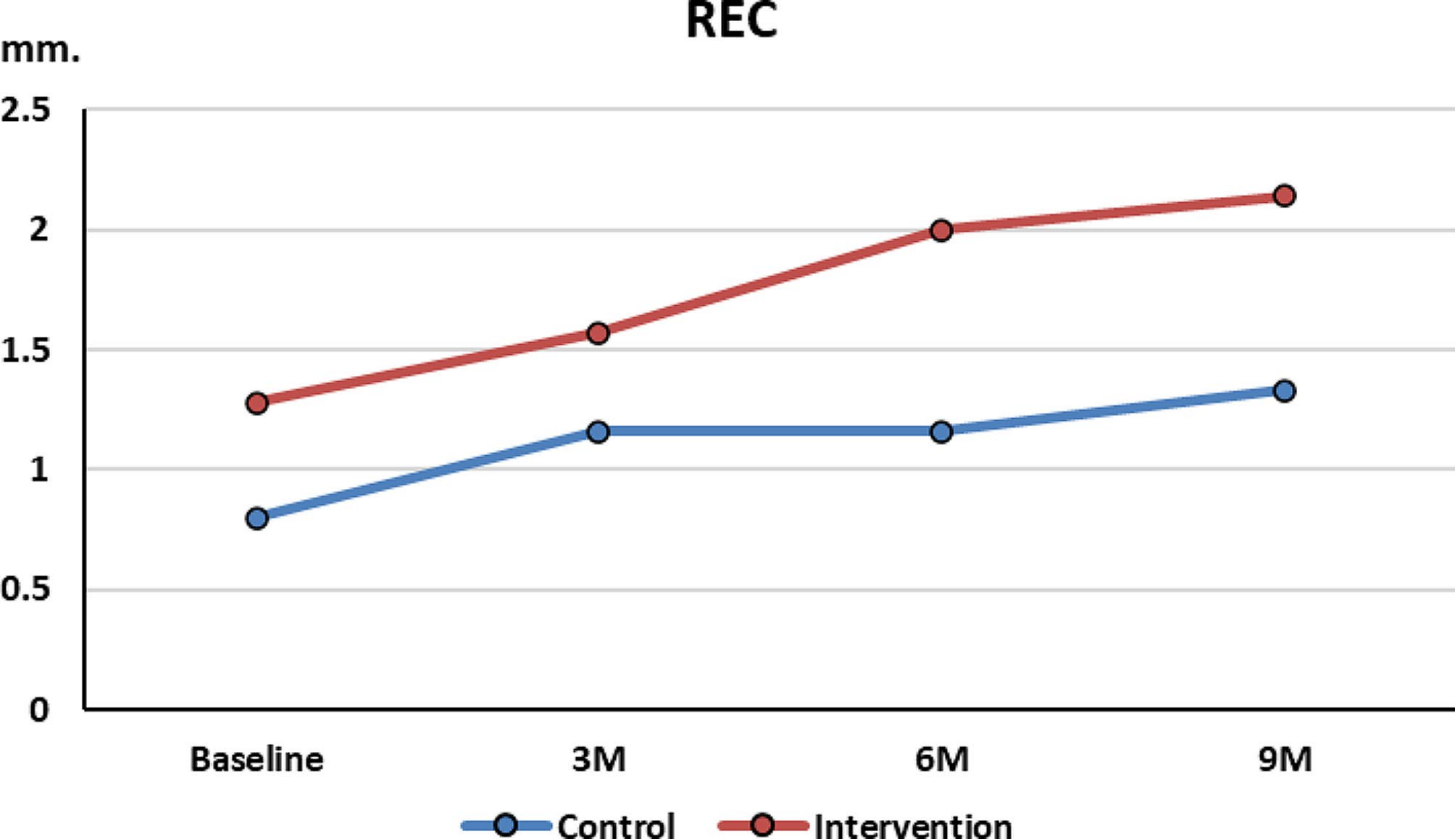
Recession changes over time between the 2 groups

Recession readings at baseline, were less in the control group (0.83 ± 1.3 mm) compared to the test group (1.28 ± 1.38 mm). At 3 months, Rec. increased in both groups. Less recession detected at 6 and 9 months in control group (0.33 ± 0.21 mm, *p* = 0.175) (0.5 ± 0.34 mm, *p* = 0.203) compared to the test group (0.71 ± 0.286 mm, *p* = 0.047) (0.857 ± 0.26 mm, *p* = 0.017) respectively (Fig. 8).

### 4- Angle of the defect measurement

At baseline, the angle of the defect was recorded in control group (46.3 ± 14) and test group (47.9 ± 19.7) with no statistically significant difference between the groups.

At 6 months, there is an increased angle of the defect control group reaching (52.93 ± 12) followed by a further increase at 9 months (59.98 ± 11, *p* = 0.001). While test group, only a slight decrease was detected at 6 and 9 months measuring (44.2 ± 14.75) (46.1 ± 16.36, *p* = 0.707) respectively.

### 5- Defect depth (DD) measurement

At baseline, DD was less in control group (2.2 ± 1.2 mm) than in test group (2.77 ± 2.3 mm) with no statistically significant difference.

At 6 and 9 months, DD increased in control group (0.38 ± 0.2 mm) (0.68 ± 0.287 mm) respectively. While in test group, a decrease in DD at 6 months (0.193 ± 0.4 mm) followed by an increased DD (0.59 ± 0.5 mm).

### 6- CEJ to base of the defect (BD) distance

At baseline, CEJ to BD distance was in control group (6.52 ± 2.1 mm) and in test group (6.66 ± 1.68 mm). After 9 months, a reduction in CEJ to BD in control group (0.037 ± 0.4 mm, *p* = 0.936) and in test group (0.897 ± 0.6 mm, *p* = 0.184).

### 7-Pain

More than 70% of the patients in both groups reported mild pain in the first 2 days with pain levels dropping to zero level by the 7th day.

All results showed no statistically significant difference (*p* > 0.05) between the 2 groups (Table 2).

**Table 2:** Shows the clinical and radiographic outcomes between the 2 groups at 3, 6 and 9 months

Outcomes	Group (*N*)	3 months	6 months	9 months
CAL	Test	6.57 ± 0.98, *p* = 0.016	6.4 ± 1.5, *p* = 0.025	6 ± 1.9, *p* = 0.062
	Control	5.5 ± 1.5, *p* = 0.023	4.83 ± 1.47, *p* = 0.003	4.67 ± 1.6, *p* = 0.009
RPD	Test	5 ± 1.155,*p = 0.013*	4.4 ± 0.79,*p* = 0.006	3.86 ± 1,*p* = 0.017
	Control	4.33 ± 0.8,*p* = 0.008	3.66 ± 0.5,*p* = 0.002	3.33 ± 0.8,*p* = 0
REC.	Test	1.57 ± 1.27,*p* = 0.172	2 ± 1.4,*p* = 0.047	2.14 ± 1.46,*p* = 0.017
	Control	1.167 ± 1.17,*p* = 0.175	1.16 ± 1.17,*p* = 0.175	1.33 ± 1.2,*p* = 0.203
Defect angle	Test	-	44.2 ± 14.75,*p* = 0.357	46.1 ± 16.36,*p* = 0.707
	Control	-	52.93 ± 12,*p* = 0.005	59.98 ± 11,*p* = 0.001
Defect depth	Test	-	2.96 ± 1.2,*p* = 0.678	2.8 ± 1,*p* = 0.914
	Control	-	1.84 ± 0.69,*p* = 0.146	1.5 ± 0.58,*p* = 0.064
CEJ TO BD.	Test	-	-	7.56 ± 1.57,*p* = 0.184
	Control	-	-	6.56 ± 2.2,*p* = 0.936

## Discussion

Currently, the primary goals of periodontal treatment have evolved beyond arresting disease progress and moved into restoring the lost tissues, by true regeneration [27, 28]. Despite the effectiveness of surgical therapy in improving clinical periodontal parameters, a limited amount of true regeneration has been shown to occur post surgically [9]. Fulfilling the 3 tenants of regeneration remains a challenge, accordingly. The aim of the current trial was to explore the regenerative potential of preserving a healthy proportion of GT in infrabony defects, compared to its complete removal.

In the last decade, the treatment of periodontal defects has focused on minimizing soft tissue trauma by using smaller surgical incisions and the application of minimally invasive surgical techniques (MIST). These techniques have been consistently shown to improve clinical outcomes including CAL, rPD and recession in addition to improving patient reported outcomes(Cortellini, 2007, 2009, 2012; Rees, 1995). The question however remained, which substitutes or biologics could improve true regeneration or further enhance the regenerative outcome? With findings indicating that infected GT possessed cells with embryonic stem cell markers, healthy granulation tissue presented a viable and easy source for cells and signaling molecules [11]. It also questioned the necessity of the time consuming and tedious process of complete removal of GT, affirming previous conclusions that complete removal of GTs was not critical for proper healing. as the removal of GT seemed to have no effect on the improvements in clinical parameters [10]. The current study aims to investigate the difference between complete and incomplete removal of GT assessing the regenerative and osteogenic potential of leaving supra-crestal fibers at the bottom of the defect may be beneficial(McCULLOCH, 1993; Hung et al., 2012). It evaluates the hypothesis that these fibers can act as a barrier to prevent further bone loss and lead to more clinical and bone attachment stability [32].

In the current trial, at baseline, the 2 groups had similar means in CAL, Rec. and rPD. At 3, 6 and 9 months postoperatively, significant CAL gain and rPD reduction occurred in both groups with minimal change in recession dimension. The two groups showed no statistically significant difference in outcomes(*p* > 0.05). These results suggest that the time-consuming process of complete removal of GT had no benefit in improving clinical outcomes.

The results of the current study both in CAL gain and rPD at 6 months were slightly lower in the test group than those found by Mishra [18]. Similarly our results at 9 months in CAL gain for both the control group (2.4 ± 0.58 mm, *p* = 0.009) and the test group (2 ± 0.87 mm, *p* = 0.062) were less than CAL gain obtained after 1 year using M-MIST alone (5.5 ± 1.6 mm, p ˂ 0.0001). Both groups had less reduction than Cortellini control (M-MIST alone) group which had rPD reduction after 1 year (4.4 ± 1 mm) [19] These results suggest that greater CAL and rPD before surgery have more CAL gain and rPD reduction regardless of complete granulation tissue removal. [18]

Incomplete granulation tissue removal was further investigated in periodontal granulation tissue preservation (PGTP) technique by another study and significant improvement in clinical parameters was achieved. This confirms that greater initial CAL and rPD resulted in more gain in attachment and more reduction in PD regardless of the surgical technique and the presence of GT in the defect [33]. Similarly, granulation tissue preservation technique (GTPT) evaluated the effect of preserving GT in a regenerative approach using EMD. It concluded that GT in bony defect didn’t compromise healing and regeneration but rather improves CAL gain than dissecting GT from the defect [34].

Regarding recession, an increase in recession was noted in both the control and test group at 6 months. These results were in line with Mishra using M-MIST alone (0.55 ± 0.52 mm). However, the results at 9 months, exhibited more recession in both groups than reported by Cortellini control group (M-MIST Alone) after 1 year (0.3 ± 0.7 mm) [18, 19] Partial removal of GT seemed affect the stability of the gingival margin increasing recession. This hypothesis was in line with results reported by……………. It was found that osseous resective surgery (ORS) resulted in more tissue rebound (less recession) than fiber retention. Cleaning the bony defect from all tissues particularly the fibers zone in the depth of the defect lead to less recession than leaving the fibers zone [35]. This explains why our test group had higher recession rates than our control, Mishra (M_MIST) alone and Cortellini (M-MIST alone) groups [18, 19]. Despite these results, no statistical significance existed between the 2 groups.

The radiographic evaluation of bone gain, in the current study, suggest that deeper infrabony defects have more bone gain (more regenerative potential) than shallow ones [36].

At 1 year, Cortellini et al. reported bone fill about 77% ±19% in their trial with M-MIST alone. Whereas our control group had linear bone gain (0.68 ± 0.287 mm, p 0.064) compared to some bone resorption in test group (-0.59 ± 0.5, *p* = 0.914) after 9 months [19]. Cortellini (M-MIST alone) group started by deeper defects INFRA (5.2 ± 1.1) at baseline, hence, had much better linear gain compared to shallower DD (< 3 mm) in our both groups [36].

The results of the current study seem to suggest that removing granulation tissue in intra-bony defects has no benefit in healing and regeneration of lost tissues. Meticulous case selection and difficult accessibility during surgery in both groups especially in the control (complete removal of GT) were one of the biggest limitations in this study.

## Conclusion

This study showed no difference clinically and radiographically between complete removal of GT compared to incomplete or partial removal of GT in healing following M-MIST. No statistical significance difference was detected between the 2 groups.

Further studies with large sample sizes are needed to confirm these results.

## Data Availability

The data that support the findings of this study are available from the corresponding author (A.A.I.)
